# Biocompatibility and antibacterial properties of ordered PLA fiber membranes fabricated via electrospinning technology

**DOI:** 10.1016/j.isci.2026.117353

**Published:** 2026-09-08

**Authors:** Donghe Xue, Zhen Zhang, Zeyu Zhang, Xiaohu Zheng, Jialong Xu, Yao Chen, Zuo Liu, Junwen Gu, Hao Lu, Kun Li, Longwei Lu

**Affiliations:** 1 Orthopedic Center, Xiuzhou District People’s Hospital, Jiaxing, Zhejiang 314015, China; 2 Department of Biotechnology and Bio Medicine, Yangtze Delta Region Institute of Tsinghua University, Jiangxing 314006, Zhejiang, China

**Keywords:** electrospinning technology, nanofiber membranes, biocompatibility, Schwann cells, antibacterial

## Abstract

Peripheral nerve injury remains a major clinical challenge owing to its limited self-regeneration capacity and high risk of postoperative infection. In this study, highly ordered poly(lactic acid) (PLA) nanofiber membranes were fabricated via electrospinning and loaded with 2.0% moxifloxacin to achieve the dual functions of axonal guidance and localized antibacterial activity. Morphological characterization revealed uniform fiber diameters, parallel orientation, and interconnected porosity, with favorable mechanical properties and an appropriate hydrophilic-hydrophobic balance. *In vitro* assays demonstrated Schwann cells maintained sustained proliferation on the membrane surface and exhibited pronounced alignment along the fiber orientation. Antibacterial evaluations showed that the moxifloxacin-loaded ordered PLA membranes achieved nearly complete eradication of bacteria within 2 h, with the mechanism closely associated with disruption of bacterial membrane structures and induction of reactive oxygen species (ROS) accumulation. This work provides a design strategy and experimental basis for multifunctional scaffold materials integrating nerve repair and antibacterial functions.

## Highlights

• Ordered PLA nanofibers guided Schwann cell alignment and proliferation
• Moxifloxacin-loaded membranes eradicated bacteria within 2 h
• Multifunctional membranes combined nerve guidance with antibacterial activity

## Graphical abstract

**Figure:**
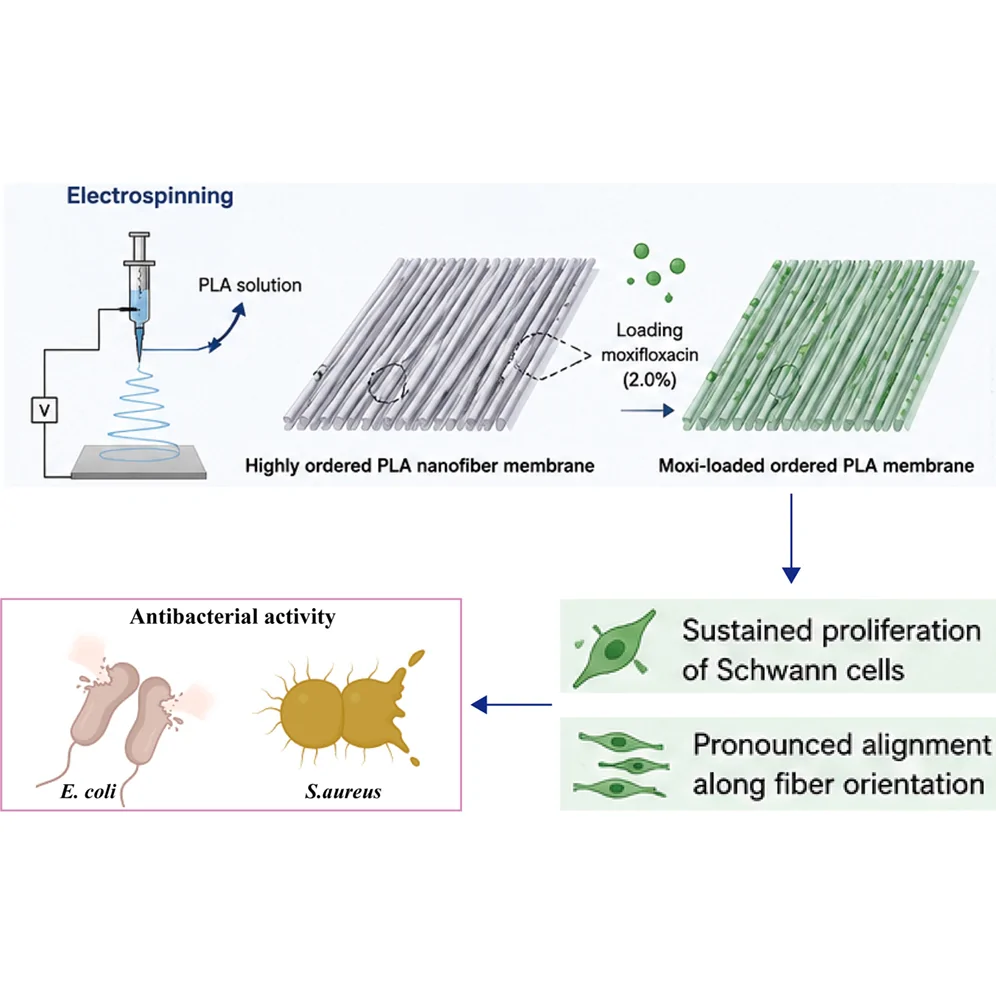

## Teaser

Biological sciences; Materials in biotechnology; Nanomaterials; Biomedical materials; Engineering materials

## Introduction

Peripheral nerve injury is a common traumatic condition in clinical practice, with sciatic nerve injury representing one of the most severe forms.^1^^,^^2^ Such injuries can lead to motor, sensory, and autonomic dysfunction, profoundly impairing the quality of life of patients.^3^ Although peripheral nerves possess a certain capacity for regeneration, spontaneous recovery is often difficult to achieve in cases involving substantial nerve defects. Traditional autologous nerve grafting remains the gold standard for repair but is limited by donor scarcity, graft atrophy, and postoperative scar formation, which constrain its clinical applicability.^4^ Peripheral nerve damage can result from trauma, high-energy impact, surgical resection, or nerve compression, and it is especially prevalent in high-risk populations such as traffic accident victims and occupational injury patients.^1^^,^^5^ Given the complex anatomy and considerable length of the sciatic nerve, its intrinsic regenerative potential is restricted.^6^ Conventional suturing techniques often fail to achieve satisfactory functional restoration in large-gap injuries,^7^ underscoring the urgent need for more effective and safer strategies for nerve repair in the field of regenerative medicine.

In recent years, tissue engineering has provided new avenues for peripheral nerve repair. Among these, the development of artificial nerve conduit materials with excellent biocompatibility and guidance capability has emerged as a major research focus; these materials serve as scaffolds to bridge nerve stumps and promote axonal regeneration.^8^ Synthetic polymers such as poly(lactic acid) (PLA) and polycaprolactone (PCL) are widely used owing to their biocompatibility, biodegradability, and processability by electrospinning. Blending PCL with natural polymers like gelatin (PCL/Gel) can further enhance bioactivity and support neurite outgrowth.^9^ Decellularized extracellular matrix (d-ECM)-based materials provide native biochemical cues that promote Schwann cell migration and axonal regeneration; however, due to their mechanical properties, they often require combination with synthetic scaffolds.^10^ Among various candidates, PLA has shown great promise in nerve tissue engineering owing to its favorable biodegradability, biocompatibility, and mechanical properties.^11^ *In vivo*, PLA undergoes hydrolytic degradation into lactic acid, which is eventually metabolized into carbon dioxide and water, and it has been widely applied in absorbable sutures, drug delivery systems, and tissue-engineered scaffolds.^11^^,^^12^ However, conventional randomly oriented PLA scaffolds have limitations in directing orderly axonal growth, which is a critical process for ensuring accurate axonal extension toward the distal end of the injury site. To address this limitation, electrospinning technology has been extensively used in recent years to fabricate nanofiber or microfiber membranes with highly ordered arrangements.^13^^,^^14^ This technique employs a high-voltage electrostatic field to stretch polymer solutions into fine fibers, which are subsequently deposited onto a collector to form a fibrous network. By controlling the configuration of the collector and the distribution of the electric field, highly aligned fibers can be obtained, which mimic the structural characteristics of the native nerve tissue and facilitate Schwann cell migration and axonal extension along specific directions.^15^^,^^16^ Numerous studies have confirmed the effectiveness of ordered electrospun fibers in peripheral nerve repair. Previous research shows that ordered nanofibers can regulate Schwann cell orientation, promote neurite extension, and enhance axonal regeneration in *in vitro* and *in vivo* experiments. Studies have found that compared with random fibers, ordered electrospun nanofibers significantly promote neurite extension.^16^ Furthermore, ordered PLA nanofibers can effectively guide neurite growth along the fiber axis.^17^ In recent years, electrospun scaffolds incorporating extracellular matrix components or immobilized growth factors have also been developed to further enhance nerve regeneration.^9^ These studies fully demonstrate the important role of topological cues in nerve repair.

Nevertheless, bacterial infection remains one of the major factors compromising the efficacy of nerve conduits in clinical applications.^18^ Nerve tissue, once damaged and surgically exposed, is highly susceptible to bacterial contamination, particularly from common pathogens such as *Staphylococcus aureus* (*S. aureus*) and *Escherichia coli* (*E. coli*), which can form biofilms on scaffold surfaces, triggering persistent local inflammation, hindering tissue regeneration, and in severe cases necessitating scaffold removal.^18^^,^^19^ Traditional systemic antibacterial therapy often fails to achieve therapeutically effective drug concentrations at the local site and can contribute to the development of drug resistance. Moxifloxacin is a novel fourth-generation fluoroquinolone antibiotic characterized by a broad antimicrobial spectrum, high efficacy, and low toxicity.^20^^,^^21^ It is widely used in the treatment of respiratory tract infections, urinary tract infections, and postoperative surgical infections, and exhibits strong inhibitory activity against common pathogens such as *S. aureus*, *E. coli*, and *Streptococcus pneumoniae* (*S. pneumoniae*).^21^^,^^22^ Although moxifloxacin has been incorporated into local drug delivery systems, including hydrogel patches and wound dressings, its application in the field of nerve repair has not been extensively explored.^23^ From a tissue engineering perspective, postoperative infection and insufficient directional guidance for axonal regeneration remain two major limitations of existing nerve conduits. Directionally aligned electrospun fibers can provide physical cues that guide Schwann cell alignment and migration, which are critical for axonal regeneration, whereas localized delivery of antibacterial agents can effectively reduce infection risk and create a favorable microenvironment for nerve repair. Therefore, the development of ordered PLA fiber scaffolds with both nerve regeneration-promoting and antibacterial capabilities holds promise for overcoming the limitations of conventional nerve conduits, which are often structurally simple and lack effective antimicrobial properties. Many electrospun neural repair scaffolds still rely on multicomponent systems, surface-immobilized bioactive molecules, or extracellular matrix derivatives to enhance bioactivity. While these strategies are effective, they often enhance the complexity of material preparation, cost, and potential translational difficulties. Moreover, while *in vivo* experiments are crucial for clinical translation, *in vitro* evaluation is of great significance in the early stages of the development of neural repair materials. *In vitro* experiments allow for the systematic evaluation of the cell compatibility of a material, cell-directed guidance capabilities, and drug release behavior under controlled conditions. This evaluation helps elucidate the material-cell interaction mechanisms and provides an important design basis for subsequent *in vivo* experiments. Therefore, *in vitro* studies are an important screening and optimization step before complex animal experiments. Based on this rationale, the present study is aimed at fabricating highly ordered PLA nanofiber membranes via electrospinning, with moxifloxacin loading to impart antibacterial activity. The biocompatibility, cellular guidance effects, and *in vitro* antibacterial performance of the materials were systematically evaluated to provide both theoretical and experimental foundations for the development of multifunctional scaffolds for peripheral nerve injury repair, particularly addressing the challenge of simultaneously preventing postoperative infection and promoting tissue regeneration.

## Results

### Macroscopic and microscopic structural characteristics of electrospun ordered PLA fiber membranes

**Figure 1:**
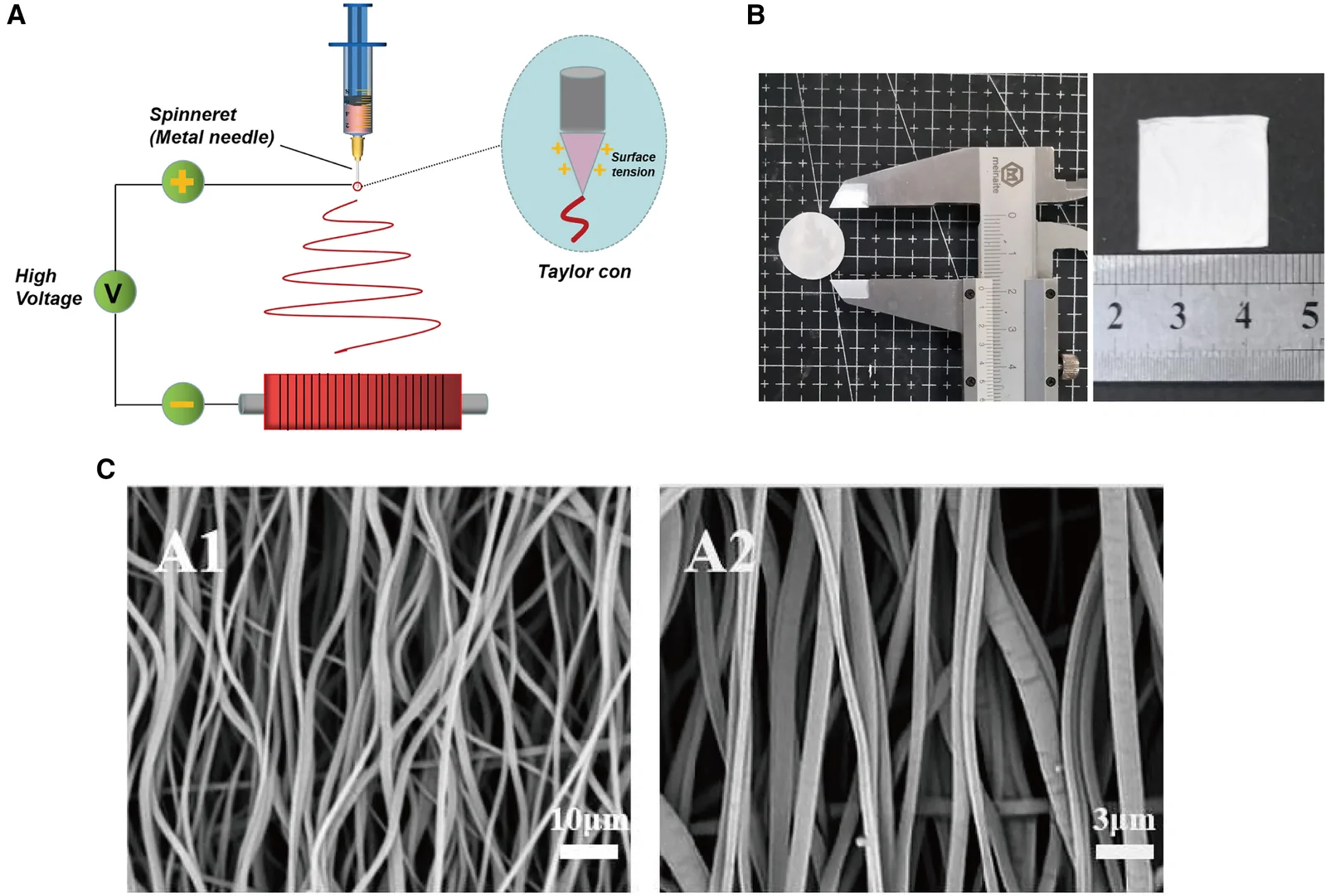
Fabrication and structural characterization of ordered PLA electrospun fiber membranes (A) Schematic of electrospinning showing Taylor cone formation and deposition onto a high-speed rotating collector to produce aligned fibers. (B) Macroscopic appearance of uniform, smooth PLA membranes. (C) SEM images showing densely packed, parallel-aligned fibers with uniform diameters and an interconnected porous structure. The scale for A1 is 10 μm, and the scale for A2 is 3 μm.

In this study, the fabrication process and structural characteristics of the electrospun fiber membranes were systematically characterized, and the electrospinning principle was illustrated via the formation of a Taylor cone under a high-voltage electric field. The process led to continuous fiber deposition on a rotating collector (Figure 1A). Macroscopic observation revealed that the resulting PLA membranes were white, opaque, and uniform thin sheets with smooth surfaces and regular edges (Figure 1B). They could be cut into various shapes and sizes as well as exhibited excellent film-forming properties and processability to meet the requirements for scaffold integrity and stability in subsequent biological experiments. At the microscopic level, scanning electron microscopy (SEM) revealed the orientation and multiscale morphological features of the membranes (Figure 1C). Low-magnification images showed fibers that were predominantly aligned in parallel along a uniform direction, densely packed, and free of bead defects (Figure 1C). Fiber diameters were evenly distributed, reflecting a stable and reproducible electrospinning process. The fibers formed interconnected pores of varying shapes and sizes, including irregular polygonal pores and elongated slits along the fiber orientation (Figure 1C), with good structural continuity to facilitate three-dimensional (3D) cell infiltration and nutrient exchange. High-magnification images showed smooth fiber surfaces with well-defined contours. Multiple fiber layers were interlaced along the thickness direction, forming a compact and 3D network (Figure 1C). In some areas, the pores exhibited elongated axes consistent with the fiber alignment. This ordered, porous 3D architecture supported stable cell adhesion and migration, and guided Schwann and other nerve-related cells for oriented axonal growth. It also provided ample sites for loading and releasing bioactive agents like moxifloxacin, enabling both nerve repair and localized antibacterial functions. Collectively, this fabrication method and resulting membrane architecture established a robust structural and morphological foundation for the application of ordered PLA fiber membranes in nerve tissue engineering and antimicrobial scaffold development.

### Effects of PLA concentration on fiber morphology and diameter distribution

**Figure 2:**
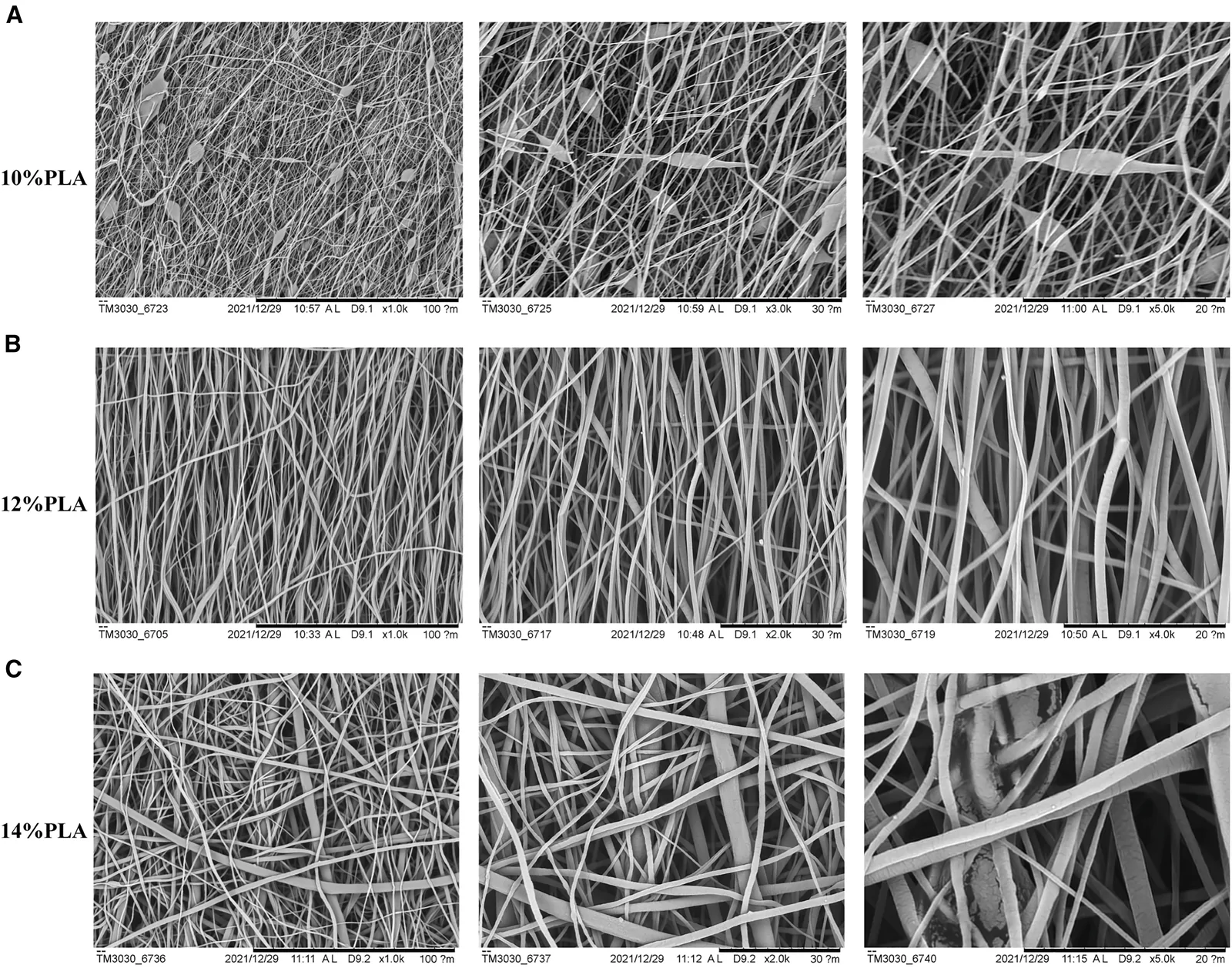
SEM analysis of electrospun PLA fiber membranes prepared at different concentrations SEM images of PLA membranes at 10% (A), 12% (B), and 14% (C) concentrations captured at different magnifications. The image scale bars are 100 μm, 30 μm, and 20 μm, respectively, from left to right.

The quality and structure of the electrospun ordered PLA fiber membranes were critically affected by the polymer concentration. PLA solutions at 10%, 12%, and 14% were prepared, and their membranes were characterized by SEM to assess their alignment, surface integrity, and pore structure. At 10% PLA, the fibers showed bead defects, uneven diameters, rough surfaces, and loose networks due to low viscosity and poor jet stability (Figure 2A). At 12% PLA, the fibers were uniformly aligned, smooth, and defect-free with consistent diameters and evenly distributed pores, forming a dense 3D network ideal for mechanical support, cell adhesion, migration, and drug diffusion (Figure 2B). At 14% PLA, the fibers were thicker, uneven, and partially fused or collapsed, with reduced porosity and slightly degraded alignment (Figure 2C). Diameter analysis confirmed a significant increase with concentration. The fiber diameter increased significantly with increasing PLA concentration, showing statistically significant differences among the three groups (Figure S1). Moreover, incorporating gelatin into 12% PLA broadened the fiber diameter distribution, caused curling and fusion, increased the surface roughness, and reduced the alignment; these effects could be attributed to the altered viscosity, surface tension, and solidification dynamics (Figure S2). Although gelatin improved the hydrophilicity, it compromised the structural uniformity and orientation. Therefore, pure 12% PLA ordered membranes were chosen for further biocompatibility and antibacterial evaluations.

### Characterization of the properties of the ordered PLA fiber membranes

**Figure 3:**
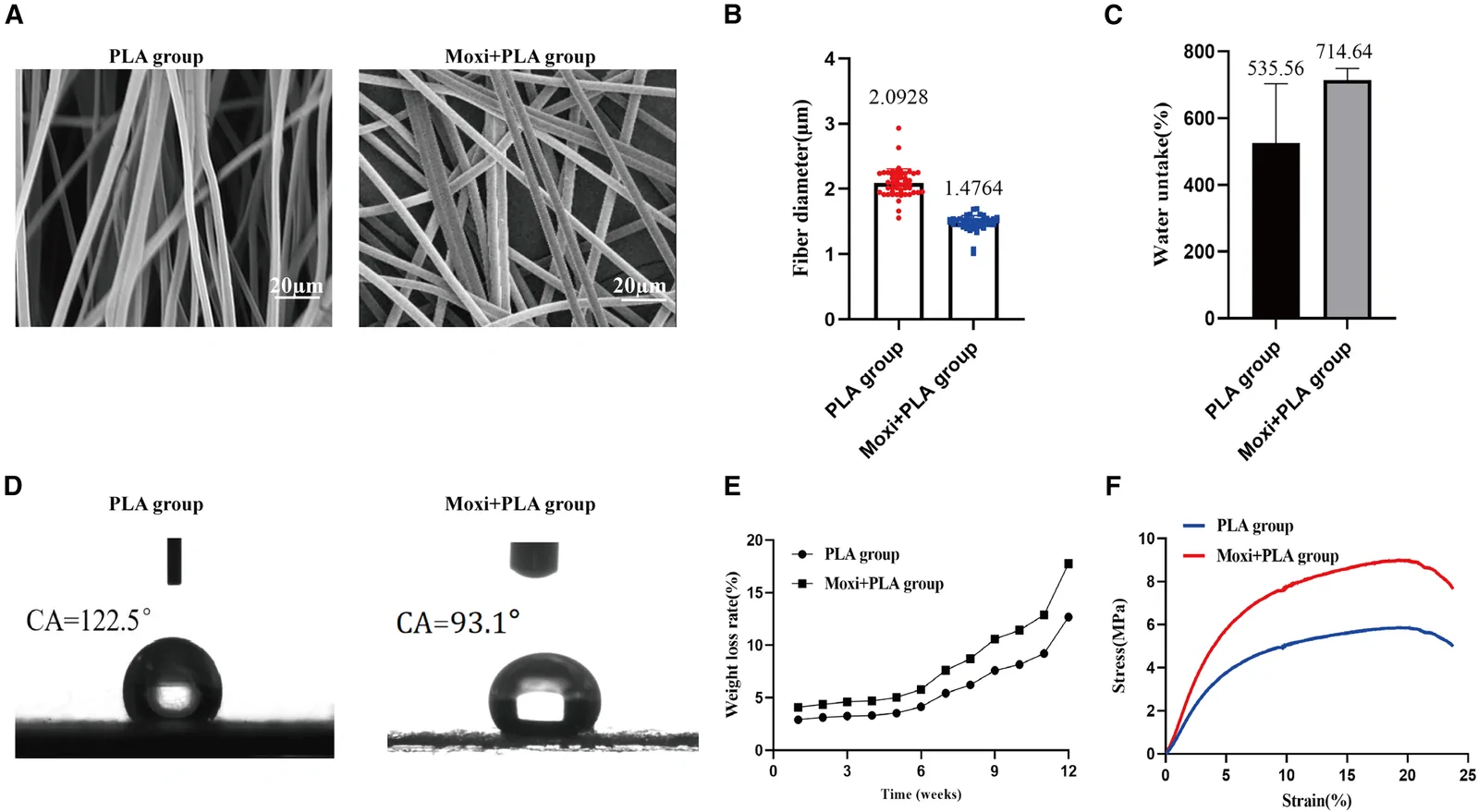
Physicochemical characterization of PLA and Moxi-loaded PLA electrospun fiber membranes (A) SEM images of electrospun PLA fibers and Moxi+PLA fibers. The image scale bar is 20 μm. (B) Quantitative measurement of fiber diameter distribution (*n* = 50 fibers per group). (C) Water uptake measurement of fiber membranes (*n* = 5 independent samples per group). (D) Water contact angle measurement of fiber membranes. (E) *In vitro* degradation behavior evaluated by weight loss ratio over time. (F) Tensile stress-strain curves of fiber membranes. Data are presented as mean ± SD (*n* ≥ 3).

Following optimization of the fabrication parameters and structural morphology of the ordered PLA fiber membranes, their physicochemical properties were systematically characterized to assess their suitability for nerve repair applications. After drug loading, the fiber morphology changed (Figure 3A). Compared with the blank fibers, the drug-loaded fibers showed a reduced average diameter. In addition, some fibers exhibited surface attachments and a less smooth surface, indicating that drug incorporation affected the stability of the spinning solution. Partial color changes of the fibers were also observed, which provided preliminary visual evidence of successful drug loading (Figure 3A). Compared with the unloaded PLA fiber membrane, the drug-loaded fibers showed a more uniform diameter distribution and maintained good continuity, although the average diameter decreased. Statistical analysis showed that the average diameter of the blank PLA fibers was 2.09 ± 0.21 μm, whereas that of the drug-loaded PLA fibers was 1.47 ± 0.15 μm (Figure 3B). Water absorption tests showed that the Moxi+PLA fiber membrane showed a significantly higher water uptake (714.64%) than did the PLA group (535.56%), indicating that drug incorporation improved the hydrophilicity of the material. The water contact angle results were consistent with this finding (Figure 3C). The contact angle decreased from 122.5° ± 6.1° in the PLA group to 93.1° ± 1.5° in the Moxi+PLA group, demonstrating enhanced surface wettability (Figure 3D). Degradation tests showed that both the groups exhibited similar degradation rates at the early stage. However, with prolonged incubation, the Moxi+PLA group exhibited a slightly higher weight loss rate, suggesting that the drug may promote material hydrolysis or increase water penetration (Figure 3E). Mechanical testing showed that the overall mechanical performance of the Moxi+PLA fiber membrane was better than that of the pure PLA membrane (Figure 3F). It exhibited higher stress-bearing capacity and improved elongation ability. Overall, these properties provided both a stable structural support and a favorable cellular microenvironment, laying the groundwork for subsequent biocompatibility and antibacterial performance studies.

### Ordered PLA fiber membranes exhibited good biocompatibility and promoted directional growth of Schwann cells

**Figure 4:**
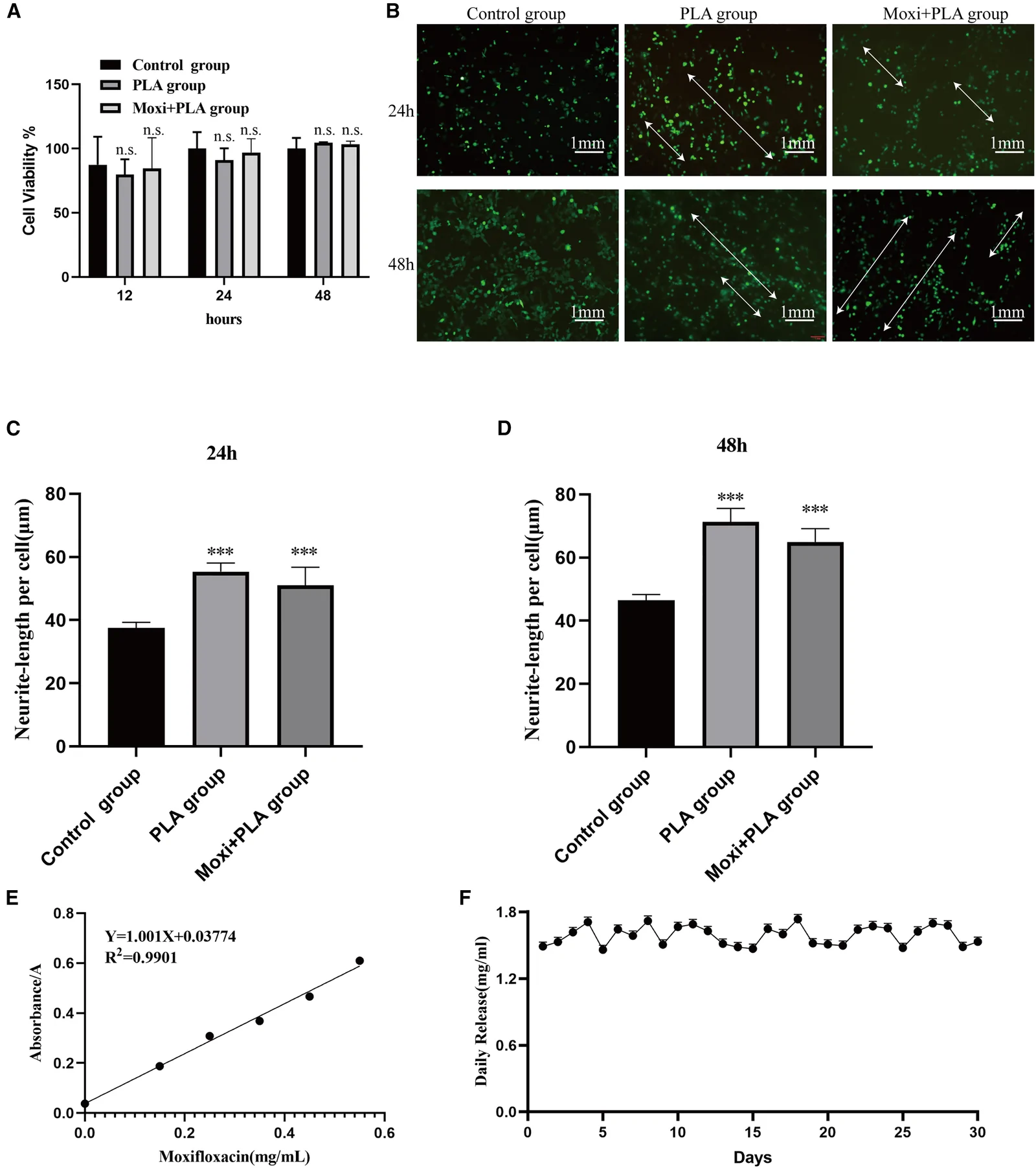
Effects of ordered PLA fiber membranes on Schwann cell proliferation and morphology (A) CCK-8 assay analysis of Schwann cell proliferation in a preconditioned medium obtained from the ordered PLA membranes and in a standard culture medium. (B) Microscopic images of RSC96 cells cultured on tissue culture plates and ordered PLA fiber membranes. The image scale bar is 1 mm. (C) Quantitative analysis of neurite length per cell at 24 h (*n* = 20). (D) Quantitative analysis of neurite length per cell at 48 h (*n* = 20). (E) Standard curve for moxifloxacin quantification based on absorbance measurements. (F) Daily release profile of moxifloxacin from Moxi+PLA fiber membranes over 30 days. Data are presented as mean ± SD (*n* = 3). At least three biological replicates were obtained for the data. Statistical analysis was performed using one-way ANOVA followed by Tukey’s post hoc test. n.s., not significant; ∗∗∗*p* < 0.001.

To evaluate the neurotoxicity and neural tissue compatibility of the ordered PLA fiber membranes, RSC96 Schwann cells were transduced with Green Fluorescent Protein (GFP)-expressing lentivirus and assessed using two approaches. Firstly, CCK-8 assay results showed that cell viability increased over time at 12, 24, and 48 h. No significant differences were observed among the groups, indicating that drug loading did not cause obvious adverse effects on cell viability. In all the groups, the cells maintained a high survival rate throughout the culture period. The optical density (OD) values increased significantly over time and maximized at 48 h, suggesting continuous cell proliferation (Figure 4A). This result suggested that during immersion, the membranes did not release toxic substances that could impair cell activity, confirming good biocompatibility and the absence of cytotoxicity (Figure 4A). Secondly, when RSC96 cells were directly cultured on the surface of the ordered PLA membranes, fluorescence microscopy revealed that at 24 h, the cells in both the groups were evenly distributed, spindle-shaped, or elongated, with clearly extended cell bodies (Figure 4B). In the PLA and Moxi+PLA groups, the cells displayed pronounced orientation, with most aligned along the fiber direction. By 48 h, the cell density had further increased, resulting in the formation of compact intercellular networks (Figure 4B). The cells in the PLA group showed more continuous distribution along the membrane surface and more uniform alignment, exhibiting distinct directional growth (Figure 4B). Quantitative analysis of the neurite outgrowth showed that at both 24 h (Figure 4C) and 48 h (Figure 4D), the neurite length in PLA and Moxi+PLA groups was significantly higher than that in the control group. This result indicates that the ordered fiber structure can promote Schwann cell process extension. Meanwhile, drug incorporation showed minimal influence on neurite outgrowth. The drug quantification standard curve showed a strong linear relationship between absorbance and moxifloxacin concentration (R^2^ = 0.9901), indicating good accuracy and reliability of the detection method (Figure 4E). Drug-release kinetics results showed that the Moxi+PLA drug-loaded fiber membrane maintained a relatively stable daily release rate over 30 days (Figure 4F). No obvious burst release was observed, suggesting good sustained release performance of this system. These findings demonstrated that the ordered PLA fiber membranes were nontoxic to the Schwann cells, provided a favorable substrate for adhesion and proliferation, and possessed strong potential for neural tissue compatibility and directional guidance.

### Ordered PLA nanofiber membranes loaded with 2.0% moxifloxacin exhibit broad-spectrum and potent antibacterial activity

**Figure 5:**
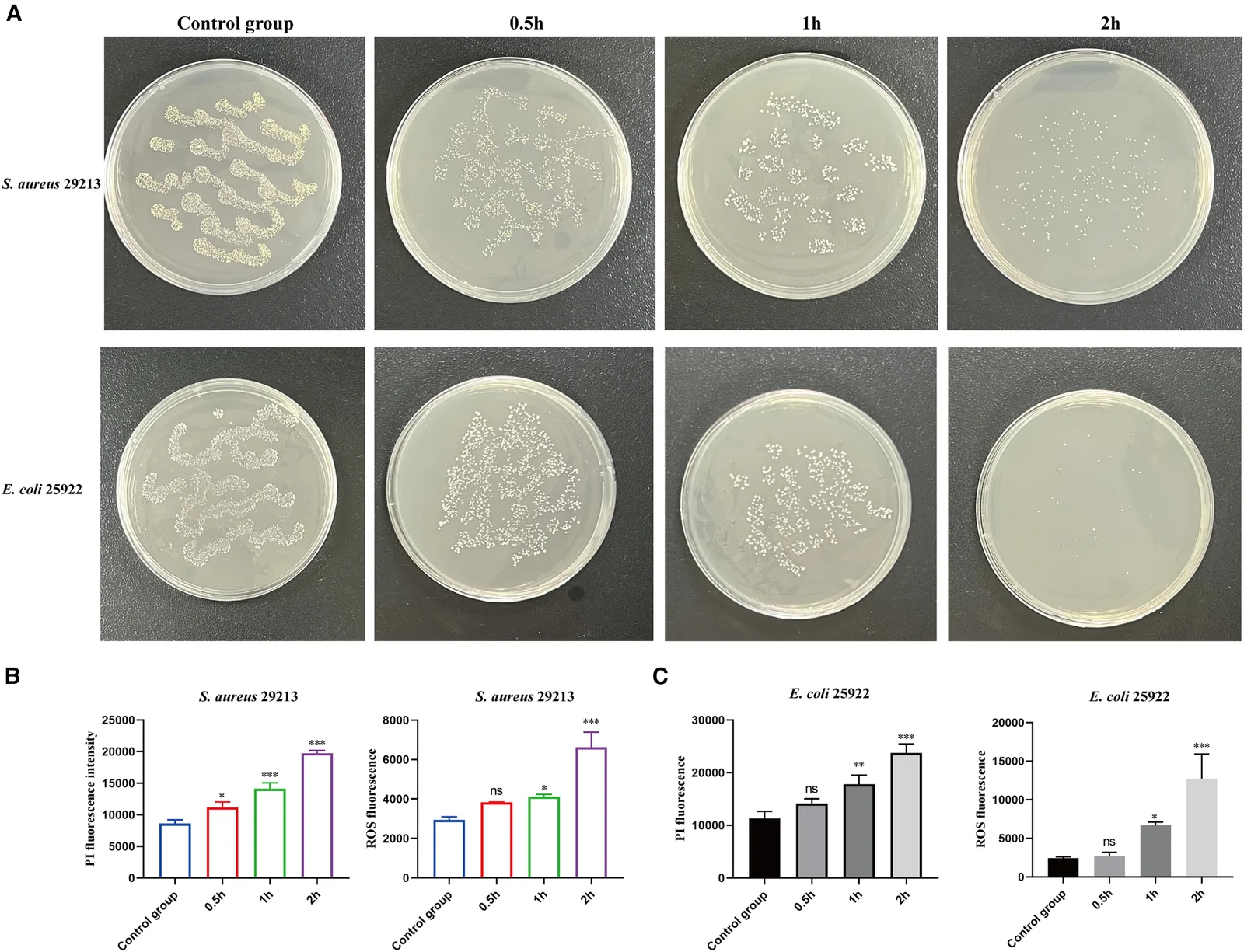
Antibacterial activity of ordered PLA nanofiber membranes loaded with 2.0% moxifloxacin (A) Colony formation of *S. aureus* 29213 and *E. coli* 25922 after treatment for different time intervals. Changes in bacterial membrane integrity (PI fluorescence) (B) and ROS levels (C) over time. Data are presented as mean ± SD (*n* = 3). Statistical analysis was performed using one-way ANOVA followed by Tukey’s post hoc test. At least three biological replicates were obtained for the data. n.s., not significant; ∗*p* < 0.05, ∗∗*p* < 0.01, ∗∗∗*p* < 0.001.

To evaluate the antibacterial performance and elucidate the potential action mechanism, the ordered PLA nanofiber membranes loaded with 2.0% moxifloxacin were tested against the Gram-positive *S. aureus* 29213 and Gram-negative *E. coli* 25922. Antibacterial assays showed pronounced time-dependent bactericidal effects against both the bacteria. Compared to the control group, the colony counts were markedly reduced at 0.5 h, further decreased at 1 h, and became nearly zero by 2 h, indicating that the membranes could rapidly and efficiently kill bacteria (Figure 5A). Bacterial membrane integrity analysis revealed a significant increase in propidium iodide (PI) fluorescence over time, indicating progressive loss of membrane integrity. In *S. aureus* 29213, the PI fluorescence increased significantly at 0.5 h, followed by increasing further at 1 and 2 h (Figure 5B). In *E. coli* 25922, the PI fluorescence increased significantly at 1 h, followed by maximizing at 2 h (Figure 5C). ROS assays demonstrated that in *S. aureus* 29213, the ROS levels were comparable to those in the controls at 0.5 and 1 h; however, they increased significantly at 2 h (Figure 5B). In *E. coli* 25922, the ROS levels were significantly elevated at 1 h and further increased at 2 h (Figure 5C). These findings suggested that the antibacterial mechanism involved membrane disruption accompanied by ROS accumulation. SEM imaging of the bacterial morphology on the membrane surface supported these observations. In the control group, the bacteria were densely distributed, with intact cell contours, smooth surfaces, and typical rod-shaped morphology (Figure S3). In contrast, the moxifloxacin-loaded membrane group exhibited markedly fewer bacteria, with the remaining cells showing pronounced shrinkage, collapse, rupture, surface irregularities, and fragmentation, which indicated severe structural damage (Figure S3). Together, these results confirm that the ordered PLA nanofiber membranes loaded with 2.0% moxifloxacin can rapidly and effectively kill both Gram-positive and Gram-negative bacteria through a mechanism involving disruption of membrane integrity, leakage of intracellular contents, and oxidative damage.

## Discussion

Peripheral nerve injury repair requires scaffold materials that provide structural support for axonal regeneration as well as address the high risk of postoperative infection, which can compromise repair outcomes.^18^ Conventional nerve conduits often fall short in meeting both requirements simultaneously—random fiber orientation limits their capacity to direct axonal growth,^24^ and the absence of inherent antibacterial properties leaves the repair site vulnerable to microbial colonization, particularly by *S. aureus* and *E. coli*, which can form persistent biofilms.^19^ Recent advances in electrospinning technology have enabled the fabrication of highly ordered nanofiber scaffolds that mimic the aligned architecture of the native nerve tissue, offering physical guidance cues for Schwann cell migration and axonal extension. Incorporating antibacterial agents into such scaffolds represents a promising strategy to combine regenerative and anti-infective functions within a single implantable material.^15^^,^^25^ In this study, an ordered PLA nanofiber membrane was successfully fabricated via electrospinning, and antibacterial functionality was achieved by loading moxifloxacin. The structural morphology, physicochemical properties, biocompatibility, cell-guiding ability, and *in vitro* antibacterial activity of the material were systematically evaluated. The results demonstrated that this multifunctional scaffold design offered advantages, particularly in simultaneously providing structural guidance and localized antibacterial effects.

In terms of morphological and structural optimization, highly aligned PLA fiber membranes were achieved this study by precisely controlling the electrospinning parameters, thereby overcoming the limitations of randomly oriented fibers in axonal guidance. Previous studies have shown that nerve axon regeneration relies heavily on the orientational cues provided by the extracellular matrix, whereas randomly distributed scaffolds often result in dispersed axonal growth, thereby reducing the functional recovery efficiency.^26^^,^^27^ The 12% PLA fiber membranes fabricated in this study exhibited orderly parallel alignment, a uniform diameter distribution, and a well-interconnected porous architecture under an SEM device; these features facilitate the directional migration of Schwann cells as well as provide a structural basis for drug loading and release.^28^ Compared with common nonaligned PLA membranes,^29^^,^^30^ this ordered architecture maintains its mechanical performance while optimizing the microenvironment for cell behavior regulation. The physicochemical properties of the membranes also demonstrated notable advantages. With a water absorption rate exceeding 525%, the membranes can maintain a stable moist environment at the nerve repair site, which is important for cellular metabolism and axonal regeneration. Contact angle measurements revealed moderate surface hydrophobicity (∼122°)—a surface property distinct from that obtained via conventional hydrophilic modification strategies^31^; this “externally hydrophobic, internally hydrophilic” profile minimizes rapid fluid dispersion while retaining internal moisture. Furthermore, the membranes displayed a stable degradation profile (12.68% weight loss over 12 weeks), ensuring structural integrity during the critical phases of nerve regeneration and preventing early scaffold collapse. The maximum tensile strength was higher than that of the rat sciatic nerve (2.7 MPa),^32^ indicating that the scaffold combined flexibility with load-bearing capacity—this is an especially important feature for peripheral nerve repair materials subjected to tensile forces during movement. The tensile properties of the electrospun fiber membranes were measured under dry-state conditions to ensure testing stability. However, mechanical properties under hydrated conditions are more relevant for *in vivo* applications because in these conditions, materials are exposed to physiological fluids after implantation. Therefore, future studies will further evaluate the mechanical behavior of the membranes under wet-state conditions to better simulate physiological environments.

In terms of biocompatibility and cell behavior, the results obtained in this study verified the directional guidance effect of the ordered PLA membranes on the Schwann cells. CCK-8 assays showed no apparent cytotoxicity, with cells maintaining a steady proliferation trend over 48 h. Fluorescence microscopy further confirmed that the Schwann cells extended along the fiber alignment and formed dense intercellular networks. This outcome is consistent with those of previous reports on oriented PCL or collagen scaffolds promoting Schwann cell migration.^33^^,^^34^ Similarly, in the present study, comparable guidance effects were achieved within a pure PLA system, without the need for additional bioactive molecule modification. Such a purely physical orientation-guidance strategy not only simplifies fabrication but also reduces biosafety risks. In terms of antibacterial functionality, the novelty of this study lies in directly incorporating moxifloxacin into ordered PLA nanofiber membranes to achieve the synergistic effect of structural guidance and localized antimicrobial action. Compared with common surface-coating antibacterial strategies,^35^^,^^36^ this approach enables uniform drug distribution within the fibers for sustained release, avoiding rapid depletion or wash-off of surface-bound agents. The results demonstrated that ordered PLA membranes loaded with 2.0% moxifloxacin could reduce *S. aureus* and *E. coli* colony counts to nearly undetectable levels within just 2 h, exhibiting broad-spectrum and highly efficient bactericidal activity. This killing rate is markedly faster than that of some silver nanoparticle- or quaternary ammonium salt-modified membranes.^37^^,^^38^^,^^39^ Mechanistic exploration indicated that the antibacterial effect was closely related to disruption of the bacterial membrane integrity and induction of ROS accumulation. PI staining and ROS assays showed that the moxifloxacin-loaded membranes significantly enhanced the bacterial membrane permeability and ROS levels at different time points, ultimately leading to cell collapse and rupture. This observation aligns with the known mechanism of fluoroquinolones, which disrupt DNA gyrase and topoisomerase IV activity, triggering oxidative stress.^40^^,^^41^ SEM observations of bacterial shrinkage and rupture further supported this synergistic mechanism. These findings suggest that integrating antibacterial agents into oriented scaffolds physically hinders bacterial adhesion as well as rapidly eradicates residual bacteria at the chemical level, thereby substantially reducing the risk of implant-associated infection. Notably, the current study primarily focuses on evaluating antibacterial efficacy and material-related performance, rather than direct molecular-level validation. Future studies involving gene expression analysis of DNA replication-related genes or enzymatic activity assays of DNA gyrase and topoisomerase IV would help further elucidate the detailed antibacterial mechanism of the drug-loaded scaffold system.

The highlight of this study is the simultaneous realization of axonal guidance and localized antibacterial activity within a single material system, overcoming the limitations of conventional nerve repair scaffolds that are weak in bacterial infection.^19^ Clinically, patients with peripheral nerve injury often face both tissue defects and postoperative infection risks.^42^^,^^43^ Traditional approaches typically require additional antibacterial dressings or systemic antibiotics, which increase the treatment complexity and risk of antimicrobial resistance.^44^^,^^45^^,^^46^^,^^47^ The proposed strategy enables long-term antibacterial protection upon scaffold implantation, simplifying postoperative management and potentially reducing complication rates.

### Limitations of the study

Firstly, antibacterial performance has been verified primarily through *in vitro* experiments, which do not enable comprehensive evaluations in animal infection models to assess local drug release kinetics, antibacterial persistence, and tissue repair quality. Secondly, the release profile of moxifloxacin requires further optimization through *in vitro* release assays and pharmacodynamic studies to ensure effective concentrations throughout the nerve regeneration process. Future research could also explore coloading with other bioactive molecules, such as neurotrophic factors, to achieve synergistic effects on nerve regeneration and immune regulation.

## Resource availability

### Lead contact

Requests for further information and resources should be directed to and will be fulfilled by the lead contact, Longwei Lu (lulongwei2@163.com).

### Materials availability

This study did not generate new unique reagents.

### Data and code availability

• The datasets generated during this study have been deposited in Mendeley Data and are publicly available as of the date of publication. The accession information is listed in the key resources table. https://doi.org/10.17632/d3jt683z8d.1.
• This paper does not report original code.
• Any additional information required to reanalyze the data reported in this paper are available from the lead contact upon request.

## Acknowledgments

This work was supported by grants from the Zhejiang Province Traditional Chinese Medicine Science and Technology Plan Project [2025ZX034].

## Author contributions

L.L. and Zhen Zhang, conceptualization; D.X., K.L., and Zeyu Zhang, methodology; D.X., X.Z., J.X., and Y.C., validation; Z.L., J.G., and H.L., formal analysis; L.L. and Zhen Zhang, writing – original draft and writing – review and editing. All authors reviewed the manuscript and approved the final version of the manuscript.

## Declaration of interests

The authors declare no competing interests.

## STAR★Methods

### Key resources table

**Table:** 

REAGENT or RESOURCE	SOURCE	IDENTIFIER
**Bacterial and virus strains**		

*S. aureus*	ATCC	ATCC29213
*E. coli*	ATCC	ATCC 25922
pCDH-CMV-EF1-copGFP lentivirus	School of Basic Medical Sciences, Zhengzhou University	N/A

**Chemicals, peptides, and recombinant proteins**		

Polylactic acid	Sigma-Aldrich	Cat#38534
Moxifloxacin	MCE	Cat#HY-66011A
Dichloromethane	Sinopharm	Cat#40013561
N,N-dimethylformamide	Sinopharm	Cat#40072363
Gelatin	Sigma-Aldrich	Cat#1288485
Phosphate-buffered saline	Gibco	Cat#20012027
Fetal bovine serum	Gibco	Cat#A5256701
Dulbecco’s modified Eagle medium	Gibco	Cat#31800022
Propidium iodide	Beyotime	Cat#C1008S
2′,7′-dichlorodihydrofluorescein diacetate	Beyotime	Cat#S0033S

**Critical commercial assays**		

CCK-8 assay kit	Beyotime	Cat#C0038

**Deposited data**		

Raw and analyzed data	Mendeley Data	https://doi.org/10.17632/d3jt683z8d.1

**Experimental models: Cell lines**		

RSC96 cells	ATCC	CRL-2765

**Software and algorithms**		

GraphPad Prism 9.0	https://www.scienceplus.com/nl/prism-commercieel.html	N/A
ImageJ software	National Institutes of Health (NIH)	RRID: SCR_003070

### Method details

#### Materials and reagents

Polylactic acid (PLA, molecular weight ≥100 kDa) was purchased from Sigma-Aldrich (USA); moxifloxacin (purity ≥98%) was obtained from MCE (China); dichloromethane (DCM) and N,N-dimethylformamide (DMF) were purchased from Sinopharm Chemical Reagent Co., Ltd. (Shanghai, China); gelatin (Type B) was obtained from Sigma-Aldrich (USA). The CCK-8 assay kit was purchased from Beyotime Biotechnology (Shanghai, China); phosphate-buffered saline (PBS) was obtained from Gibco (USA); fetal bovine serum (FBS) and high-glucose Dulbecco’s modified Eagle medium (DMEM) were purchased from Gibco (USA). Propidium iodide (PI) and 2′,7′-dichlorodihydrofluorescein diacetate (DCFH-DA) were purchased from Beyotime Biotechnology (Shanghai, China). *S. aureus* (ATCC 29213) and *E. coli* (ATCC 25922) were obtained from the American Type Culture Collection (ATCC). All reagents were of analytical grade or cell culture grade.

#### Fabrication of ordered PLA nanofiber membranes

Based on previously reported methods with minor modifications.^48^ All spinning experiments were performed inside a fume hood under strictly controlled environmental conditions to ensure process stability and reproducibility. The laboratory temperature was maintained at 24°C ± 3°C, and the relative humidity was controlled at 50 ± 5% RH, as environmental fluctuations can significantly affect solvent evaporation and fiber morphology. For solution preparation, dichloromethane (DCM) and N,N-dimethylformamide (DMF) were mixed at a volume ratio of 2:1 to prepare 21 mL of mixed solvent. Polylactic acid (PLA, M_w_ ≈ 200 kDa, NatureWorks, USA) was gradually added to obtain a spinning solution with a final concentration of 12% (w/v). The solution was magnetically stirred at room temperature for 4 h until completely dissolved and homogeneous. The solution was then allowed to stand for 30 min to remove air bubbles and subsequently filtered through a 0.45 μm hydrophobic membrane to remove particulates and residual bubbles. The prepared spinning solution was loaded into a 10 mL syringe equipped with a metallic needle (inner diameter 0.6 mm, 22G). The needle tip was connected to the positive terminal of a high-voltage power supply set at 21 kV. The syringe was mounted on a syringe pump, and the solution was delivered at a constant feeding rate of 0.05 mm/s. The distance between the needle tip and the collector was fixed at 17 cm. A high-speed rotating drum collector covered with aluminum foil was used to collect fibers. The drum rotation speed was set at 2000 rpm to obtain aligned fiber structures. During electrospinning, the formation of the Taylor cone and jet stability were continuously monitored, and spinning was maintained under stable conditions until membranes with the desired area and thickness were obtained. After electrospinning, the fiber membranes were carefully peeled from the collector and placed in a desiccator for light-protected drying. The membranes were then stored in sealed packaging for subsequent experiments. Under the optimized and fixed electrospinning parameters described above, three independent batches of samples were prepared to ensure process stability and reproducibility.

#### Preparation of moxifloxacin-loaded ordered PLA fiber membranes

Moxifloxacin was added to the 12% PLA solution at a final concentration of 2.0% (w/w) and magnetically stirred until uniformly mixed. The drug-loaded solution was then electrospun under the same conditions described above to obtain moxifloxacin-loaded fiber membranes. After drying, the membranes were cut into 15 mm diameter disks or 10 × 10 mm squares and stored under sterile conditions until use.

#### Morphological and structural characterization

The samples were sputter-coated with a ∼20 nm gold layer under vacuum to enhance conductivity and observed using a scanning electron microscope (SEM, TM3030, Japan) at an accelerating voltage of 5 kV. Surface morphology at the nanoscale was obtained through the interaction between the electron beam and the sample surface, allowing assessment of fiber uniformity and formation stability. Fiber diameters were measured by randomly selecting fibers from high-magnification images using ImageJ software, and the resulting data were used to generate diameter distribution histograms to evaluate diameter uniformity and fabrication reproducibility.

#### Measurement of water absorption of PLA fiber membranes

Following previously reported methods with minor modifications,^49^ the prepared fiber membrane samples were dried to a constant weight in a vacuum desiccator and weighed (dry mass, m_d_) using an electronic balance with a precision of 0.1 mg. The samples were then fully immersed in deionized water and incubated at room temperature (∼25°C) in the dark for 48 h to allow complete penetration of water into the porous structure. After soaking, the samples were gently removed with tweezers, and excess surface water was carefully blotted with lint-free filter paper without applying pressure to avoid deformation. The wet mass (m_w_) was measured immediately to minimize errors from evaporation. Water absorption was calculated using the formula: Water uptake (%) = (m_w_–m_d_)/m_d_ × 100%, and the mean value ± standard deviation was calculated.

#### Water contact angle analysis

Following previously reported methods with slight modifications,^50^ the wettability of the fiber membrane surface was characterized at room temperature (∼25°C) using a contact angle goniometer (JC2000C, POWE-REACH Technology Co., Ltd.). Before testing, the fiber membrane samples were flatly fixed onto glass slides to avoid wrinkles or additional tension. At least six independent parallel samples were prepared for each experimental group. Ultrapure water (resistivity ≥18.2 MΩ cm) was used as the testing liquid. The droplet volume was strictly controlled at 5 μL using a precision microsyringe to ensure consistent droplet size and gravitational effects across all measurements. During measurement, the droplet was slowly released from the needle onto the sample surface. Images were captured by the high-speed camera of the instrument within 5 s after the droplet contacted the sample surface. To minimize the influence of local surface heterogeneity, at least five random positions were measured on each sample (*n* = 6 independent samples, total of 30 measurement points). The contact angle values were automatically calculated using the built-in Young–Laplace fitting algorithm. The final data were expressed as the mean value ± standard error of the mean (SEM) of all measurement points.

#### Degradation performance assessment

Following previously reported methods with slight modifications,^51^ ordered PLA fiber membranes were cut into curled shapes matching the form of nerve conduits, and their initial dry weight (W_0_) was recorded. The samples were rinsed with deionized water, dried, and placed into tubular fiber membranes, then submerged in sterile PBS buffer in 120 mm diameter culture dishes. Blank PBS dishes were used as controls. All samples were incubated statically at 37°C. During the degradation test, PBS was replaced weekly, and one set of samples was collected at each time point. Retrieved membranes were gently rinsed three times with deionized water to remove residual salts, blotted with filter paper to remove surface moisture, and dried in a vacuum oven for 4 h until a constant weight (W_1_) was reached. Since CO_2_ bubbles generated on the sample surface during degradation could affect the PBS environment, PBS was refreshed every three days to maintain stability. The weight loss ratio was calculated as: W_loss_(%) = (W_0_−W_1_)/W_0_ × 100%.

#### Mechanical property characterization

Following previously reported methods with slight modifications,^51^ the prepared and stabilized ordered PLA fiber membranes were cut into rectangular strips measuring 10 mm × 30 mm along the fiber alignment direction. Tensile tests were conducted at room temperature and standard atmospheric pressure using a universal testing machine (Instron 3343, Instron Ltd., USA). The effective clamping length at both ends of each specimen was set to 10 mm, and the tensile rate was maintained at 5 mm/min. Prior to testing, the thickness of each specimen was measured at three different points using a precision micrometer, and the mean value was used as the thickness parameter. The load cell of the testing machine was zeroed before each test to ensure measurement accuracy.

#### Evaluation of neurotoxicity and neural tissue compatibility of PLA fiber membranes

Following previously reported methods with slight modifications,^51^ ordered PLA fiber membranes were soaked in double-distilled water for 12 h, sterilized at high temperature, and rinsed five times with PBS buffer (3 min each). The membranes were then immersed in high-glucose DMEM containing 10% FBS and 1% penicillin–streptomycin and incubated at 37°C in a 5% CO_2_ atmosphere for 48 h to prepare PLA preconditioned medium. RSC96 cells (gifted by the School of Basic Medical Sciences, Zhengzhou University) were cultured in high-glucose DMEM supplemented with 10% FBS and 1% penicillin–streptomycin at 37°C under a 5% CO_2_ atmosphere. For subculturing, old medium was removed, cells were washed twice with PBS, and digested with trypsin for 8 min. The digestion was terminated, and the cells were resuspended by gentle pipetting and passaged at a ratio of 1:3. Cell viability was assessed using the CCK-8 assay. RSC96 cells were seeded at an appropriate density in 96-well plates and cultured with PLA preconditioned medium for 12, 24, and 48 h. At each time point, 10 μL of CCK-8 working solution was added to each well and incubated for 4 h. Absorbance (OD) was measured at 450 nm using a microplate reader. Cell viability was calculated according to the following equation: Cell viability (%) = [OD450(sample)–OD450(blank)]/[OD450(control)−OD450(blank)] × 100%. Sample, control, and blank represent the absorbance values of the treated group, untreated control group, and blank wells (medium without cells), respectively.

#### Evaluation of the guidance effect of PLA fiber membranes on cultured neural cells

The experiment included fiber membrane pretreatment and fluorescence microscopy observation of cell growth. Electrospun ordered PLA fiber membranes were cut to appropriate sizes, immersed in 3% benzalkonium bromide solution for 24 h for sterilization, rinsed thoroughly with PBS, and air-dried for later use. For lentiviral infection, logarithmic-phase RSC96 cells were seeded at a density of 2 × 10^5^ cells/well in 6-well plates. After 24 h of culture, when cell confluence reached approximately 30%, pCDH-CMV-EF1-copGFP lentivirus (MOI = 10; gifted by the School of Basic Medical Sciences, Zhengzhou University) and 40 μL of TransGP infection enhancer were added. Following 10 h of incubation, the viral supernatant was removed and replaced with fresh DMEM medium for continued culture. For guidance evaluation, pretreated fiber membranes were placed in 12-well plates, and 5 × 10^5^ GFP-labeled RSC96 cells were seeded onto each membrane. Wells without membranes served as the control group. After incubation at 37°C in a 5% CO_2_ atmosphere for the designated time, cell adhesion and growth on the fiber membrane surfaces were observed using a fluorescence microscope to assess the ordered membranes’ ability to guide and support neural cell growth.

#### *In vitro* antibacterial activity assay

Following previously reported methods with minor modifications,^52^ standard strains *S. aureus* ATCC 29213 and *E. coli* ATCC 25922 were cultured in LB broth at 37°C with shaking at 200 rpm until reaching logarithmic growth phase (OD_600_ ≈ 0.3–0.5). Bacterial suspensions were then diluted with sterile PBS to approximately 1 × 10^6^ CFU/mL. Sterile drug-loaded PLA membrane discs (15 mm diameter) were placed in contact with equal volumes of the bacterial suspension in sterile Petri dishes and incubated statically at 37°C for 0.5 h, 1 h, or 2 h. A blank control group (no membrane treatment) was included. After incubation, the reaction mixtures were spread evenly onto LB agar plates and incubated inverted at 37°C for 24 h before observing and photographing bacterial colony growth.

#### Bacterial membrane integrity assay

Following previously reported methods with slight modifications,^53^ bacterial suspensions were prepared as described above. At each designated time point, an aliquot of bacterial suspension was mixed with propidium iodide (PI) to a final concentration of 10 μg/mL and incubated at room temperature in the dark for 15 min. After incubation, samples were transferred into black 96-well plates, and red fluorescence intensity was measured using a multimode microplate reader at excitation/emission wavelengths of 535/617 nm (bandwidth set to instrument defaults). Negative controls (untreated bacterial suspension+PI) were included, and all groups were tested in triplicate. Results were expressed as mean ± SD.

#### Bacterial ROS detection

Following previously reported methods with slight modifications,^54^ logarithmic-phase bacterial cells were collected by centrifugation (5000 × g, 5 min, 4°C), resuspended in sterile PBS, and adjusted to approximately 1 × 10^6^ CFU/mL 2′,7′-Dichlorodihydrofluorescein diacetate (DCFH-DA) was added to a final concentration of 10 μM, and the suspension was incubated at 37°C in the dark with shaking for 30 min to allow probe loading. After incubation, the cells were gently centrifuged (5000 × g, 5 min), the supernatant was discarded, and the pellet was washed twice with PBS to remove excess extracellular probe. The probe-labeled bacterial suspension was then treated with the test samples (with PBS as control). At designated time points (0.5 h, 1 h, 2 h), aliquots were transferred immediately into black 96-well plates. Fluorescence intensity was measured using a multimode microplate reader at an excitation wavelength of 488 nm and an emission wavelength of 525 nm (bandwidth and sensitivity set to instrument defaults). To eliminate the effect of cell density differences, OD_600_ values were recorded simultaneously, and fluorescence readings were normalized (fluorescence intensity/OD_600_). Each experiment was performed in triplicate, and results were expressed as mean ± SD.

#### Scanning Electron Microscopy (SEM) Observation

To examine bacterial interaction with the fiber membrane surface and associated morphological changes, sterilized fiber membranes were incubated with bacterial suspensions for 2 h and then immediately fixed in 2.5% glutaraldehyde for 4 h to preserve the structural integrity of both the cells and materials. Following fixation, samples were dehydrated through a graded ethanol series (30%, 50%, 70%, 90%, and 100%), with each step lasting 15 min to ensure thorough removal of water. The dehydrated samples were dried in a critical point dryer to prevent structural collapse, sputter-coated with a ∼20 nm gold layer to enhance conductivity, and examined using a scanning electron microscope (SEM, SU8010, Hitachi, Japan) at appropriate accelerating voltages to record the surface morphology of both the materials and bacteria.

### Quantification and statistical analysis

All data were expressed as mean ± standard deviation (Mean ± SD), with each experiment performed in at least triplicate. Statistical analyses were conducted using GraphPad Prism 9.0. Differences between two groups were evaluated using a two-tailed unpaired *t* test, while comparisons among multiple groups were performed using one-way analysis of variance (One-way ANOVA). Statistical significance was defined as ∗*p* < 0.05, ∗∗*p* < 0.01, ∗∗∗*p* < 0.001, and n.s. indicated no significant difference.
