# Supplementary material for: Biocompatibility and antibacterial properties of ordered PLA fiber membranes fabricated via electrospinning technology
Source: iScience. 2026 Sep 8;29(9):117353. doi: 10.1016/j.isci.2026.117353 (PMC13573699; doi:10.1016/j.isci.2026.117353)
Supplement: Supplementary file 1 [file mmc1.pdf]

iScience, Volume *29*

Supplemental information

Biocompatibility and antibacterial properties of ordered PLA fiber membranes fabricated via electrospinning technology

Donghe Xue, Zhen Zhang, Zeyu Zhang, Xiaohu Zheng, Jialong Xu, Yao Chen, Zuo Liu, Junwen Gu, Hao Lu, Kun Li, and Longwei Lu

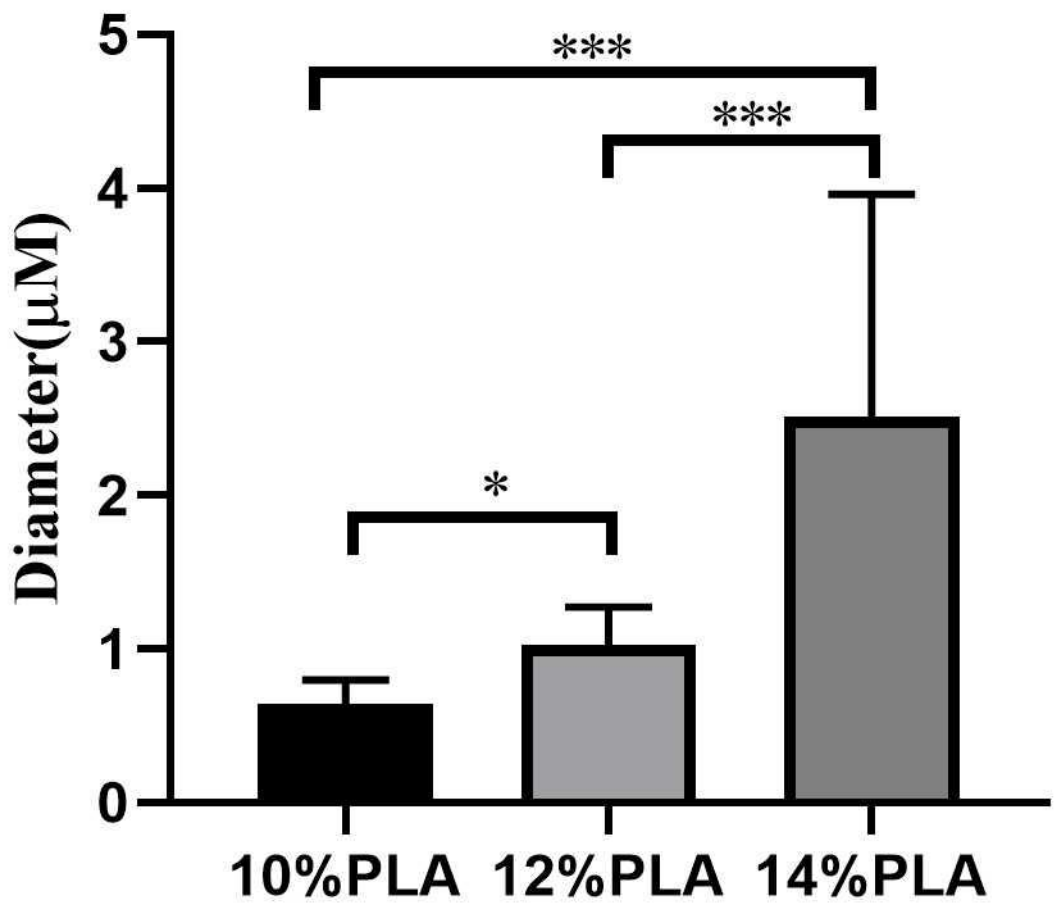

Figure S1. Quantitative analysis of fiber diameters in PLA membranes prepared at different concentrations (10%, 12%, and 14%). Fiber diameter increased significantly with PLA concentration. Data are presented as mean ± SD (n = 3). Statistical analysis was performed using one-way ANOVA. *p < 0.05, **p < 0.001.

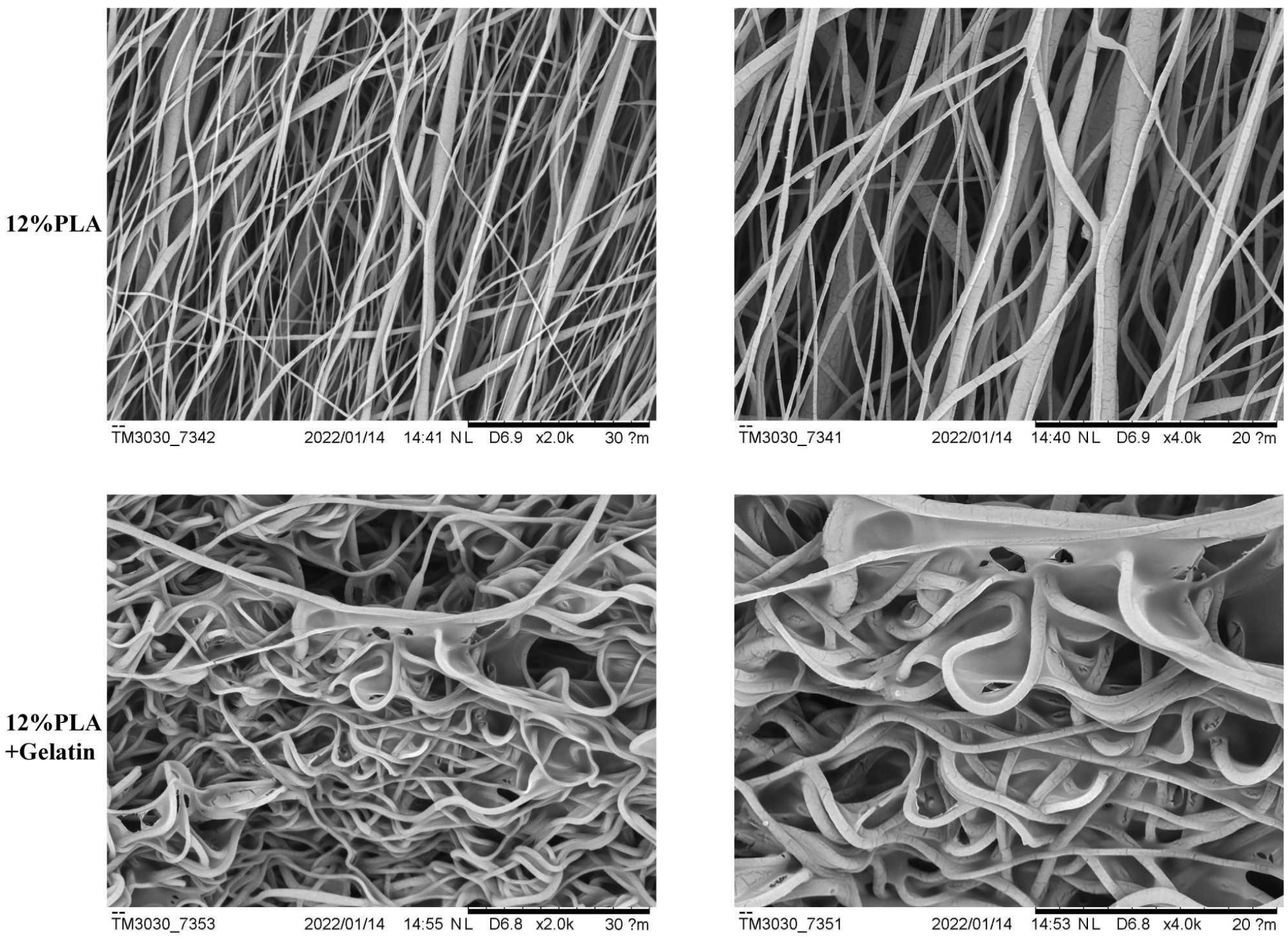

Figure S2. SEM comparison of 12% PLA and 12% PLA/gelatin composite fiber membranes. Gelatin incorporation resulted in a broader fiber diameter distribution, localized curling, fusion or sheet-like structures, increased surface roughness, and reduced alignment.The image scale bars were 30μm, and 20μm, respectively, from left to right.

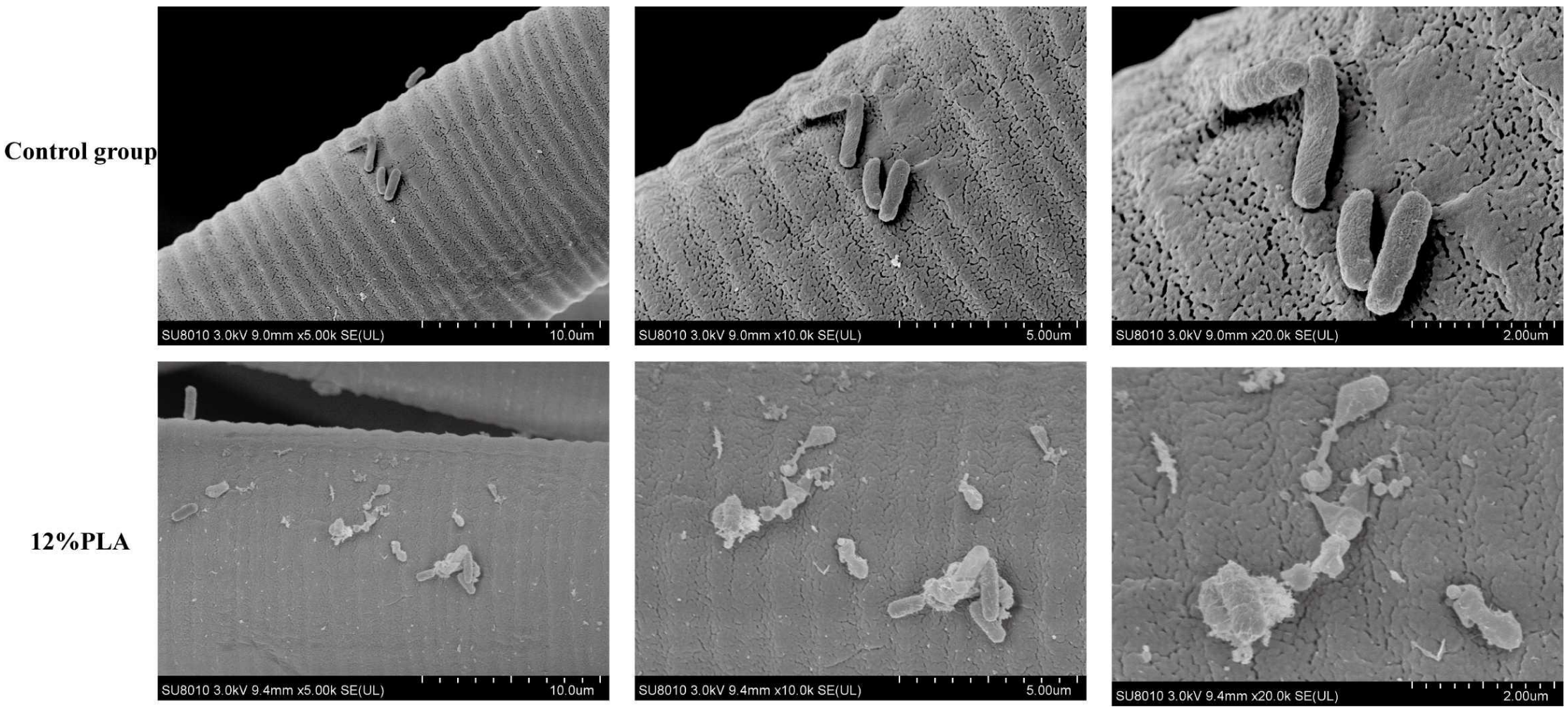

Figure S3. SEM observation of bacterial morphology on the surface of ordered PLA nanofiber membranes loaded with 2.0% moxifloxacin. The image scale bars were 10μm, 5μm, and 2μm, respectively, from left to right.
